# Ribose 5-phosphate: the key metabolite bridging the metabolisms of nucleotides and amino acids during stringent response in *Escherichia coli*?

**DOI:** 10.15698/mic2023.07.799

**Published:** 2023-06-01

**Authors:** Paulina Katarzyna Grucela, Tobias Fuhrer, Uwe Sauer, Yanjie Chao, Yong Everett Zhang

**Affiliations:** 1 Department of Biology, University of Copenhagen, DK-2200 Copenhagen, Denmark.; 2 Institute of Molecular Systems Biology, ETH Zurich, Zurich, Switzerland.; 3 The Center for Microbes, Development and Health (CMDH), Institut Pasteur of Shanghai, Chinese Academy of Sciences, Shanghai 200031, China.

**Keywords:** (p)ppGpp, stringent response, nucleotide, amino acid, ribose 5'-phosphate

## Abstract

The bacterial stringent response and its effector alarmone guanosine penta- or tetra – phosphates (p)ppGpp are vital for bacterial tolerance and survival of various stresses in environments (including antibiotics) and host cells (virulence). (p)ppGpp does so by binding to its numerous target proteins and reprograming bacterial transcriptome to tune down the synthesis of nucleotides and rRNA/tRNA, and up-regulate amino acid biosynthesis genes. Recent identification of more novel (p)ppGpp direct binding proteins in *Escherichia coli* and their deep studies have unveiled unprecedented details of how (p)ppGpp coordinates the nucleotide and amino acid metabolic pathways upon stringent response; however, the mechanistic link between nucleotide and amino acid metabolisms remains still incompletely understood. Here we propose the metabolite ribose 5'-phosphate as the key link between nucleotide and amino acid metabolisms and a working model integrating both the transcriptional and metabolic effects of (p)ppGpp on *E. coli* physiological adaptation during the stringent response.

Upon amino acid starvation, either artificially induced (e.g., by valine) or during nutrient downshift to amino acid free condition, the wild type (wt) *Escherichia coli* MG1655 strain elicits the stringent response by producing the small alarmone (p)ppGpp, globally reprogramming bacterial metabolism to tune down the synthesis of nucleotides and rRNA, and up-regulate amino acids biosynthesis [1–5] (**Figure 1A**). Despite the regulatory scheme being known for many years and studied at the levels of global transcription [1–3] and translation [6], the underlying mechanistic details and the relationship of nucleotide and amino acid metabolisms upon amino acid starvation started to unfold recently. Two different techniques, i.e., DRaCALA [7] and photocrosslinkable ppGpp-analogs based capture compound mass spectrometry [8], systematically identified novel direct binding proteins of (p)ppGpp in *E. coli*. Besides the large overlap, some novel (p)ppGpp targets were identified separately, e.g., PpnN [7], Gsk and PurF [8]. Subsequent more detailed analyses of these proteins [9, 10] have revealed unprecedented details of how (p)ppGpp regulates their molecular functions and thus reprogram global metabolism upon amino acid starvation. Here we discuss these two studies, emphasizing on the connection and potential role of ribose 5-phosphate (R5P) between nucleotide and amino acid metabolisms and propose a working model to integrate the effects of (p)ppGpp on both nucleotides and amino acids metabolism in *E. coli* upon amino acid starvation.

**Figure 1:**
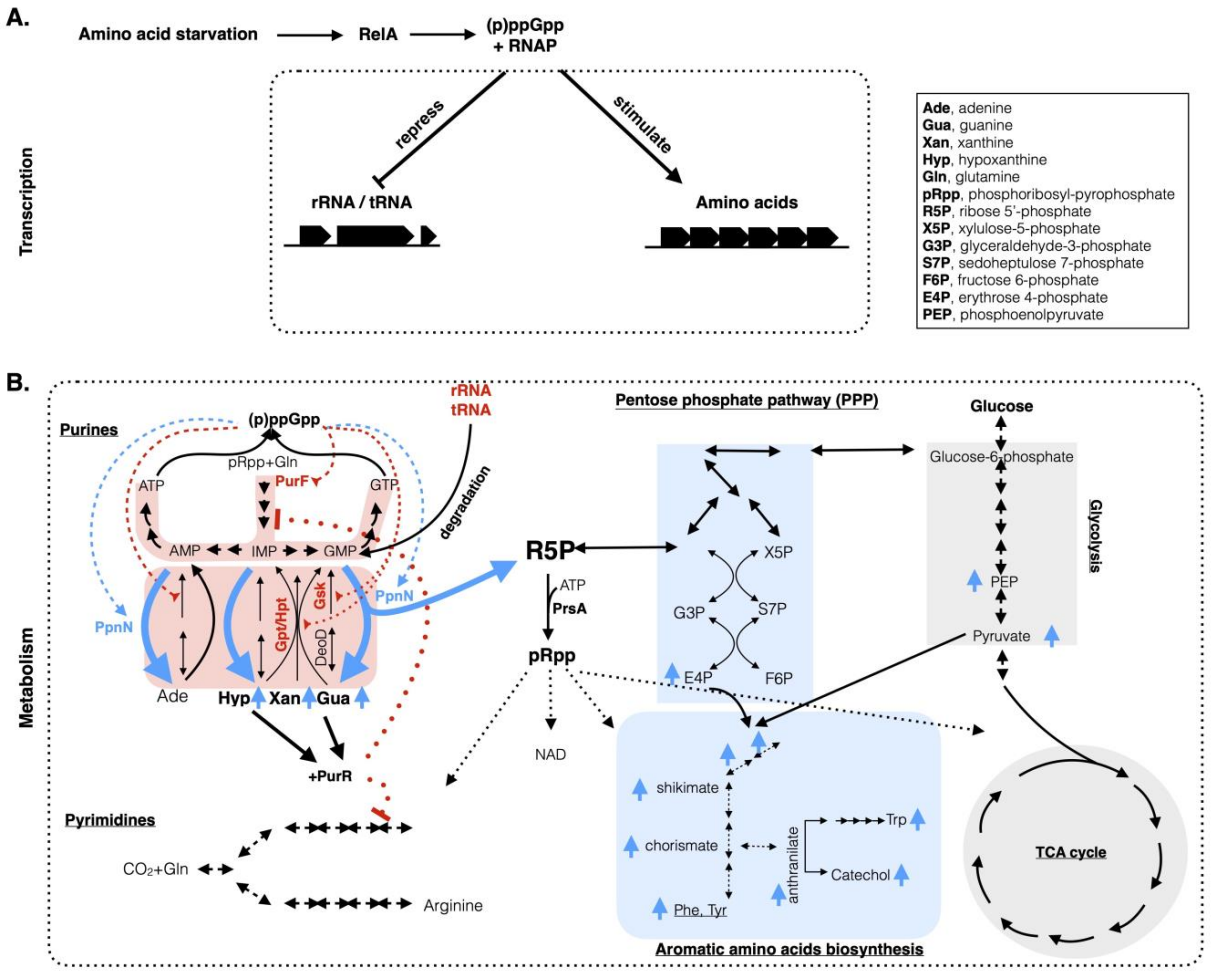
FIGURE 1: A comprehensive model integrating both the metabolic and transcriptional coordination of nucleotide and amino acid metabolisms upon stringent response in *E. coli.* **(A)** Amino acid starvation in *E. coli* leads to the activation of RelA to produce (p)ppGpp, which binds to the RNA polymerase (RNAP) to downregulate stable RNA (rRNA, tRNA) and upregulate amino acid biosynthesis genes at the transcriptional level. **(B)** At the metabolic level, (p)ppGpp directly inhibits (in red broken lines) the *de novo* and the salvage pathways (highlighted in red shadow) of purine nucleotides synthesis by targeting the PurF and Gpt/Hpt/Gsk proteins, respectively (in red font). Meanwhile, (p)ppGpp binds to PpnN (light blue font) and stimulates its activity (in blue broken lines) to degrade excess nucleotides, from both the redundant nucleotides and the degraded rRNAs/tRNAs. Degraded nucleotides lead to increased nucleobases (Gua, Xan, Hyp) (in blue up arrows) and R5P. The former ones bind to PurR to repress expression of genes (in red dotted lines) in the *de novo* purine and pyrimidine biosynthesis pathways. R5P, used to produce pRpp, enters the pentose phosphate pathway and culminates with the synthesis of (aromatic) amino acids and the intermediate metabolites. Metabolites highlighted with light blue up arrows are the ones that increased their concentrations upon amino acid starvation in the wild type *E. coli*, but not in the *ΔppnN* mutant strain [10]. The list of abbreviated metabolites is included in the inset.

The *E. coli* purine nucleotide biosynthesis pathways consist of both the *de novo* and salvage branches (**Figure 1B**, left). The *de novo* pathway starts from the formation of phosphoribosylamine from phosphoribosyl pyrophosphate (pRpp) and glutamine catalyzed by the glutamine aminophosphoribosyltransferase PurF. Subsequent nine reactions culminate with the synthesis of inosine 5'-monophosphate (IMP). From here, IMP is converted by two enzymes PurA and GuaB for the eventual biosynthesis of ATP and GTP, respectively. The salvage pathway catalyzes the one-step synthesis of purine nucleotides by transferring the R5P of pRpp to the respective nucleobases. This is catalyzed by three enzymes in *E. coli*, Gpt, Hpt, and Apt, which synthesize IMP, XMP, GMP and AMP by using the nucleobases hypoxanthine, xanthine, guanine and adenine, respectively. Additionally, the salvage pathway can be undertaken in a two-step reaction. Firstly, DeoD converts nucleobases to nucleosides, which are converted into nucleotides by Gsk. There is also a newly characterized nucleosidase PpnN which degrades nucleotides into nucleobases and R5P in a one-step reaction [11].

We recently found that during amino acid starvation (p)ppGpp allosterically stimulates the catalytic activity of PpnN to accelerate the degradation of nucleotides to nucleobases and R5P [10]. One reason underlying the stimulated PpnN activity by (p)ppGpp lies on the fact that during amino acid starvation (p)ppGpp inhibits the bulk RNA synthesis (mainly rRNA, tRNA) which consumes most of the nucleotide pool. Furthermore, stable RNA (rRNA, tRNA) is degraded during amino acid starvation [12, 13] to release more nucleotides inside cells. These two processes lead to high levels of nucleotides inside cells; however, nucleotides must be depleted to prevent resynthesis of NTPs (and ADP, see below) when cells do not need so much of them. To achieve this, besides the stimulation of PpnN, (p)ppGpp inhibits the activities of Gpt and Hpt [14] (and also Gsk [9], see below), preventing the salvage syntheses of purine nucleotides. Thus, the two opposite effects of (p)ppGpp synergistically reduce and hold the nucleotide level low upon amino acid starvation. Additionally, PpnN is ideal for this task because of its two other properties. First, PpnN is constitutively expressed inside cells and ready to be stimulated by (p)ppGpp upon amino acid starvation; but without (p)ppGpp its catalytic activity is held low [10]. Second, a sigmoidal activity curve of PpnN with the GMP substrate [10] showed that PpnN is a cooperative enzyme and more active at higher GMP levels. These properties make PpnN the ideal driver to rapidly deplete and drain the increased pool of nucleotides upon amino acid starvation. As the reaction products of PpnN, nucleobases are secreted outside cells and can be taken up and re-used for nucleotides synthesis to promote fast cell regrowth [15, 16]. However, the fate of the other reaction product R5P was not confirmed in our previous study [10], despite the obvious anticipation that R5P enters the carbon metabolic pathways.

On the other hand, the study of another (p)ppGpp target protein Gsk [9] revealed another reason why the nucleotide levels, especially ADP, must be kept low. A (p)ppGpp non-binding Gsk mutant Gsk(K383A) converts exogenous supplied nucleosides, i.e., inosine and guanosine, into nucleotides by depriving the gamma phosphate of ATP during amino acid starvation even in the presence of (p)ppGpp [9]. This reaction caused higher intracellular levels of nucleotides, especially ADP. Notably, ADP is instantly produced from the Gsk reaction and ADP binds to the essential protein PrsA to inhibit the synthesis of the crucial biosynthetic intermediate pRpp [17, 18]. pRpp is essential for the biosynthesis of pyrimidine nucleotides, and the amino acids histidine and tryptophan, and NAD etc. Consistently, supplementation with uridine, histidine and tryptophan completely suppressed the growth defects of the Gsk(K383A) mutant strain in presence of nucleosides [9]. These data suggest a key role of (p)ppGpp in balancing the metabolic pathways of purine, pyrimidine and amino acid biosynthesis pathways by lowering the nucleotides concentration and thus maintaining the production of pRpp and amino acids during amino acid starvation. However, the synthesis of pRpp in wt *E. coli* cells requires both the substrates ATP and R5P. ATP levels did not change much during amino acid starvation [9]; however, the R5P level was not shown.

Given the above analyses, we hypothesize that R5P produced from nucleotide degradation is used for the syntheses of pRpp and amino acids. We thus re-analyzed our previously published metabolomic data generated via untargeted mass spectrometry (MS) of the wt MG1655 and the Δ*ppnN* mutant strains upon valine induced amino acid starvation [10]. We found that a series of metabolites increased five minutes after the starvation in wt *E. coli* cells, but not in the Δ*ppnN* mutant. These include the pentose phosphate pathway metabolites sedoheptulose 7-phosphate (S7P), erythrose 4-phosphate (E4P), and the glycolysis metabolites phosphoenolpyruvate (PEP), pyruvate, which together contribute to the synthesis of aromatic amino acids and their biosynthesis intermediates (shikimate, chorismate; **Figure 1B**, right, in light blue up arrows). The abolished increase of these metabolites in the Δ*ppnN* mutant indicates that R5P derived from degraded nucleotides as catalyzed by PpnN substantially contributes to the synthesis of aromatic amino acids. Consistently, aromatic (and other) amino acids were higher in the wt *E. coli* than in the Gsk(K383A) mutant strain (Figure 5E, S5C in [9]) wherein the salvage pathway is uninhibited. Altogether, these data indicate that the degraded nucleotides provide (at least one source of) R5P for the synthesis of pRpp, pyrimidine nucleotides and amino acids upon stringent response.

With these, we propose a working model (**Figure 1**) integrating both transcriptional and metabolic effects of (p)ppGpp on *E. coli* stringent physiology. Upon amino acid starvation, RelA senses the uncharged tRNA at the ribosome A-site and synthesizes (p)ppGpp from GTP/GDP and ATP within several minutes. The abruptly increased (p)ppGpp on one hand binds to the RNA polymerase on two sites [19, 20] and reprogram the binding preference of RNAP [3, 21], to reduce expression of rRNA/tRNA and increase expression of amino acid biosynthesis genes. However, these amino acid synthesizing proteins need the respective precursors such as E4P which are derived from glucose catabolism but also from the intracellularly degraded rRNA/tRNA and nucleotides. Here, (p)ppGpp stimulates PpnN to degrade and deplete nucleotides, yielding nucleobases and R5P; on the other hand, (p)ppGpp inhibits further the futile cycle of re-synthesizing nucleotides via the salvage pathway enzymes Gsk, Gpt, Hpt. R5P is used to synthesize pRpp and enters the carbon metabolic pathways, which together lead to the synthesis of pyrimidine nucleotides and amino acids that are used to sustain the adapted cell growth of *E. coli*. The nucleobases can be secreted outside cells and conserved for further use during regrowth. Of note, nucleobases bind to a transcriptional repressor PurR and co-repress the expression of *de novo* synthesis genes (including PurF) [22] and in synergy (p)ppGpp directly inhibits the PurF activity [8], blocking the *de novo* pathway.

All in all, (p)ppGpp coordinates the dynamic metabolic conversions of nucleotides to amino acids via affecting both the metabolic enzyme activities and the transcriptional expression of relevant enzymes. This timely synchronization of molecular events at different levels via various target proteins of (p)ppGp ensures a prompt reprograming of cell physiology and thus a competitive ecological fitness of *E. coli* and probably many other bacteria.

## Abbreviations:

wt: – wild type.
